# Decline in Coronary Mortality in Sweden between 1986 and 2002: Comparing Contributions from Primary and Secondary Prevention

**DOI:** 10.1371/journal.pone.0124769

**Published:** 2015-05-05

**Authors:** Lena Björck, Simon Capewell, Martin O’Flaherty, Georgios Lappas, Kathleen Bennett, Annika Rosengren

**Affiliations:** 1 Department of Molecular and Clinical Medicine, Sahlgrenska Academy, Gothenburg University, Gothenburg, Sweden; 2 Institute of Health and Care Sciences, Sahlgrenska Academy, Gothenburg University, Gothenburg, Sweden; 3 Division of Public Health, University of Liverpool, Liverpool, United Kingdom; 4 Department of Pharmacology and Therapeutics, Trinity Centre for Health Sciences, St James’s Hospital, Dublin, Ireland; University of Bologna, ITALY

## Abstract

**Background:**

The relative importance of risk factor reduction in healthy people (primary prevention) versus that in patients with coronary heart disease (secondary prevention) has been debated. We aimed to quantify the contribution of the two.

**Methodology:**

We used the previously validated IMPACT model to estimate contributions from primary prevention (reducing risk factors in the population, particularly smoking, cholesterol and systolic blood pressure) and from secondary prevention (reducing risk factors in coronary heart disease patients) in the Swedish population.

**Principal Findings:**

Between 1986 and 2002, about 8,690 fewer deaths were related to changes in the three major risk factors. Population cholesterol fell by 0.64 mmol/L, with approximately 5,210 fewer deaths attributable to diet changes (4,470 in healthy people740 in patients.) plus 810 to statin treatment (200 in healthy people, 610 in patients). Overall smoking prevalence decreased by 10.3%, resulting in 1,195 fewer deaths, attributable to smoking cessation (595 in healthy people, 600 in patients). Mean population systolic blood pressure fell by 2.6 mmHg, resulting in 900 fewer deaths (865 in healthy people, 35 in patients), plus 575 fewer deaths attributable to antihypertensive medication in healthy people. The majority of falls in deaths attributable to risk factors occurred in people without known heart disease: 6,705 fewer deaths compared with 1,985 fewer deaths in patients (secondary prevention), emphasizing the importance of promoting health interventions in the general population.

**Conclusions:**

The largest effects on mortality came from primary prevention, giving markedly larger mortality reductions than secondary prevention.

## Introduction

During recent decades, age-specific coronary heart disease (CHD) mortality rates have more than halved in Sweden with similar declines in many high-income Western countries. Between 1986 and 2002, age specific CHD mortality rates in Sweden decreased by 53% in men and 52% in women aged 25–84 years [1]. However, CHD remains a leading cause of mortality in Sweden and elsewhere [2].

Using the IMPACT model, studies in the United States and Europe, including Sweden, have suggested that most of the CHD mortality fall reflects improvements in population risk factor levels [1, 3–5]. The Swedish IMPACT analysis model showed that more than half of the fall in CHD mortality rate between 1986 and 2002 was explained by changes in population risk factors, mainly reductions in cholesterol, smoking and systolic blood pressure (SBP). Treatment, including secondary prevention, explained a third of this reduction [1].

In parallel with these population risk factor changes, there have been large changes over time in medical interventions and treatments such as thrombolysis, ACE-inhibitors, beta-blockers, statins, coronary artery bypass surgery and percutaneous coronary intervention.

Health care professionals have mainly focused on medical treatments, interventions and risk factor reduction in CHD patients (secondary prevention), probably underestimating the importance of changes in population risk factors [6]. However, earlier studies have shown that primary prevention [3, 4, 7] in population may have a larger effect on CHD mortality rates than secondary prevention [8, 9]. Still, difficulties for health care professionals to see health as population problem and not only as a problem for individuals remain [6]. We therefore used the validated IMPACT model to quantify the mortality reduction between 1986 and 2002 related to secondary prevention in CHD patients and that related to primary prevention in asymptomatic individuals in the population.

## Methods

### The Swedish IMPACT model

The Excel-based Swedish IMPACT model includes all standard evidence-based medical and interventional treatments for CHD and population risk factors trends, and a ***web-based online appendix*** is available with detailed information about data sources [10]. The model was used to estimate the change in CHD mortality between 1986 and 2002, attributable to: a) individual medical and surgical treatments and interventions, and b) population risk factor trends in adults 25 to 84 years. All data were stratified by gender into 10-year age groups in patients with diagnosed CHD and individuals without known CHD.

To address the potential effect on reduction mortality (case-fatality) in individual patients receiving multiple treatments (polypharmacy) we used the Mant and Hicks cumulative relative benefit approach [11]:

$$\text{Relative benefit}\;=\;1-\prod_{S}(1-rr_{S})$$

where rr_**S**_ is the relative reduction in case-fatality rate for treatment **S** and the product is taken over all treatments.

The model has previously been used to estimate falls in CHD mortality and has been validated in different populations [3–5, 12].

### Primary and secondary prevention

Between 1986 and 2002, CHD mortality rates in Sweden fell by 53.4% in men and 52.0% in women aged 25–84 years. If the rates had remained the same as in 1986, another 13,180 CHD deaths would have occurred in 2002, in addition to the 11,850 that actually occurred. Changes in major cardiovascular risk factors (cholesterol, smoking and SBP) accounted for approximately 8,690 fewer deaths (65.9% of the 13,180 total) [1]. For the purpose of this analysis, these 8,690 fewer deaths were attributable to: a) risk factor reductions in CHD patients (secondary prevention), b) risk factor reductions in the population and c) pharmacological treatments for primary prevention.

### Data Sources

We used the IMPACT model to estimate how much of the decrease in CHD mortality in Sweden could be explained by medical treatment and by changes in major cardiovascular risk factors in the population.

Total population and age and sex distribution for Sweden in 1986 and 2002 were obtained from the National Board of Health and Welfare. Age- and gender-specific CHD mortality rates in 1986 and 2002 were obtained from the Cause of Death Register, the National Board of Health and Welfare. The number of CHD deaths expected in 2002, if mortality rates had not dropped from 1986 (baseline year) levels, was then calculated by multiplying the age- and gender-specific rates for 1986 by the population for each 10-year age stratum in the year 2002, using indirect standardization. Subtracting the number of deaths actually observed in 2002 then gave the total number of deaths prevented or postponed over the period. The number of deaths prevented or postponed because of medical treatments and interventions, and changes in population risk factors, were then calculated. A detailed description of data sources, prevalence of CHD by diagnosis, CHD incidence, case fatality by patient group, and relative and absolute risk reduction is available in earlier publications [1, 10]. Secondary prevention in CDH patients includes secondary prevention after myocardial infarction, CABG and PCI, heart failure and angina.

### Risk factors in the population

The model includes information on major CHD risk factors: mean total cholesterol level, smoking prevalence and mean SBP. Data on cholesterol were taken from the AMORIS study [13], the MONICA Gothenburg study and the INTERGENE study. Data on SBP were taken from the MONICA Gothenburg study and from MONICA Northern Sweden and the INTERGENE study. Data on smoking prevalence were obtained from the Official Statistics of Sweden.

### Risk factors and secondary prevention in CHD patients

Information on cholesterol, SBP and smoking in CHD patients was obtained from a Swedish case-control study [14] and Euroaspire II [15].

The number of CHD patients (defined as having either acute myocardial infarction or unstable angina pectoris) by diagnosis was found from the Swedish Hospital Discharge Register. The number of deaths prevented or postponed attributed to medical treatments and interventions and changes in population risk factors were then calculated. Data on secondary prevention for 2002 came from the Swedish Quality of Care Registers and Euroaspire II [15].

### Effect of population risk factor trends on CHD mortality

We used two different approaches to calculate the decrease in deaths attributable to secular trends. A regression approach was used for cholesterol and SBP. The mortality drop was calculated using:

- the number of CHD deaths in the base year 1986
- the reduction in risk factor levels between 1986 and 2002
- a regression coefficient for the change in CHD mortality per unit of absolute change in the risk factor, based on meta-analyses of large cohort studies.

#### Example 1. Regression method for SBP

In 1986 (base year), there were 570 CHD deaths among 471,039 women aged 55–64. Between 1986 and 2002, the mean SBP decreased by *D* = 2.4 mmHg. The largest meta-analysis, the Prospective Study Collaboration [16], showed an age and sex-specific reduction in mortality of 50% for every 20 mm Hg reduction in SBP, generating a logarithmic coefficient β = –0.035.

The mortality reduction was then estimated as:

$$1-e^{\left(\beta \times D\right)}\times \;deaths_{1986}=\;1-e^{\left(-0.035\times 2.4\right)}\times 570=46\;\text{fewer deaths}$$

The regression approach was repeated for men and women in all age groups. An identical approach was used for changes in cholesterol.

To determine the impact of changing prevalence of smoking, a population-attributable risk fraction approach (PARF) was used, and calculated conventionally as:

$$\text{PARF}=\frac{p\;\times \left(RR-1\right)}{p\times \left(RR-1\right)+1}$$

where *p* is the prevalence of the risk factor and RR is the relative risk of death from CHD associated with smoking. The number of deaths prevented was then calculated as the number of CHD deaths in the base year 1986 multiplied by the difference in the PARF between 1986 and 2002.

#### Example PARF method for smoking

There were 2,166 deaths from CHD in men aged 55 to 64 in 1986. The prevalence of smoking among men in this age group was 36.6% in 1986 and 24.4% in 2002. Assuming a relative risk of 2.52 [17], the PARF decreased from 0.36 to 0.27. The mortality reduction was therefore 2,166 × (0.36–0.27), or 195 fewer deaths.

### Risk factor changes in CHD patients

The mortality decrease in CHD patients due to reductions in SBP and cholesterol was calculated in a similar way and adjusted for case fatality. This was calculated using the Hospital Discharge Register and the Swedish Cause of Death Register for specific patient groups.

#### Example regression method for cholesterol

There were 18,760 male CHD patients aged 65 to 74 years, and the mean serum cholesterol decreased 0.23 mmol/L between 1986 and 2002. Their case fatality rate was 0.074. Given a relative risk reduction of 0.30, the mortality reduction was:

$$\begin{array}{c} 18,760\;\times \;0.074\;\times \;0.23\;\times \;0.30\\ =\;96\;fewer\;deaths \end{array}$$

The mortality decrease in CHD patients attributable to a reduction in smoking was calculated using the population-attributable risk fraction method described above.

### Primary preventive medication

We assessed the benefits of primary preventive medication, used to lower lipid and SBP levels. The number of deaths prevented or postponed was calculated as the product of:

- the number of eligible individuals with elevated blood pressure or cholesterol;
- the proportion who actually received the medication;
- the relative risk reduction due to the medication;
- the 1-year case fatality rate if untreated; and
- compliance (long-term adherence) with medication, which was assumed to be 50% among these asymptomatic patients in the community [18].

To avoid overlap and double counting of individuals in the IMPACT model, potential overlaps between groups were identified and appropriate adjustments made [10].

### Sensitivity analysis

All assumptions and variables were tested in a multi-way sensitivity analysis using the analysis of extremes methods. For each variable in the model, we assigned a lower value, the most likely value and an upper value, using 95% confidence intervals when available and otherwise using ± 20% [19].

## Results

Around 8,690 fewer deaths from CHD occurred in 2002 than would have been expected if the rates from 1986 not had dropped with the change attributable to changes in the three major risk factors, cholesterol, smoking and SBP (Table 1). Of these, about 6,705 deaths (77.2%) were prevented or postponed in “healthy people” (primary prevention), including from the use of statins (200 fewer deaths) and hypertension treatments (575 fewer deaths) (Table 2). About 1,985 fewer deaths (22.8%) were related to changes in risk factors and medication in CHD.

**Table 1:** Risk factor levels in CHD patients and individuals without CHD in 1986 and 2002.

Year	Risk factor	CHD patients	Total Population
**1986**			
	Cholesterol, mmol/L	6.83	6.15
	Smoking, %	32.8	28.9
	Systolic Blood pressure, mm Hg	150.9	133.8
**2002**			
	Cholesterol, mmol/L	5.20	5.51
	Smoking, %	21.1	18.6
	Systolic blood pressure, mm Hg	137.8	131.2

**Table 2:** Summary of the fall in CHD mortality attributable to risk factor changes in people with and without diagnosed CHD in Sweden 1986–2002.

		Deaths prevented or postponed^a^ (minimum and maximum estimates)		
**Risk factor**	**Absolute decrease in population risk factor level**	**Healthy individuals: primary prevention**	**CHD patients: secondary prevention**	**Totals**
**Cholesterol total change**	**0.64 mmol/L**	**4,670 (3,295–5,980)**	**1,350 (915–2,240)**	**6,020 (4,210–8,220)**
Diet		4,470 (3,225–5,430	740 (585–880)	5,210 (3,810–6,310)
Statin therapy		200 (70–550)	610 (330–1,360)	810 (400–1,910)
**Smoking**	**10.3%**	**595 (585–855)**	**600 (370–1,720)**	**1,195 (955–2,575)**
**Blood pressure total change**	**2.60 mm Hg**	**1,440 (960–1,970)**	**35 (25–130)**	**1,475 (985–2,100)**
Secular trends		865 (715–1,015)	35 (25–130)	900 (740–1,145)
Hypertension treatments		575 (245–955)	^b^	575 (245–955)
**All three major risk factors**		**6,705 (4,840–8,805)**	**1,985 (1,310–4,090)**	**8,690 (6,150–12,895)**

^a^All numbers are rounded to the nearest 5

^b^Hypertension therapy in CHD patients is already quantified within secondary prevention medication

### Cholesterol

Total cholesterol fell by 0.64 mmol/L in the whole population and by 1.63 mmol/L in CHD patients (Table 1). This large reduction in total cholesterol, from both diet and statins, resulted in an overall decrease in CHD mortality of around 6,020 fewer deaths (Table 2).

Approximately 4,670 fewer deaths or 53.7% were attributable to cholesterol decrease in “healthy individuals” in the population. A smaller proportion (<10%) of asymptomatic individuals with elevated cholesterol received statin treatment (not shown). Consequently, the effect of lipid-lowering treatment in primary prevention was low and explained only 2.3% of the reduction in mortality.

In CHD patients, around 1,350 fewer deaths or 15.5% were attributable to a decrease in cholesterol. Changes in diet explained approximately 740 fewer deaths or 8.5%, while statins explained 610 fewer deaths or 7.1%.

### Smoking

Overall smoking prevalence declined by 10.3% in the total population and by 11.7% in CHD patients (Table 1). This resulted in approximately 1,195 fewer deaths overall, out of which approximately 595 or 6.8% were attributable to reductions in asymptomatic individuals in the population and 600 fewer deaths or 6.9% were attributable to smoking reductions in CHD patients (Table 2).

### Systolic blood pressure

Mean SBP fell by 2.6 mm Hg in the total population and by 13.1 mm Hg in CHD patients (Table 1), resulting in approximately 900 fewer deaths. Less than 1% of the reduction was attributable to falls in SBP in CHD patients and around 865 or 10.0% were attributable to decreases in SBP in the total population (Table 2). Approximately 1,489,000 individuals in the population were hypertensive in 2002 [20] and about 59% received long-term medication that resulted in approximately 575 fewer deaths or 6.6%.

### Reductions in CHD mortality

#### Contribution from primary and secondary prevention by age and sex

Excluding deaths prevented by medical treatment as part of primary prevention, we can then estimate that 7,915 deaths from CHD were prevented by reductions in cholesterol, smoking and SBP, achieved by primary prevention (excluding medical treatment) and secondary prevention, including medication (Table 3). This enables us to quantify the varying effects of “whole population” changes and changes in CHD patients.

**Table 3:** CHD deaths prevented or postponed because of primary and secondary prevention by age and sex.

	CHD patients Secondary Prevention ^a^		Asymptomatic individuals Primary Prevention ^a^		Total	

Age (y)	Men n (%)	Women n (%)	Men n (%)	Women n (%)	Men n (%)	Women n (%)
25–54	50 (4.3)	70 (8.6)	430 (9.9)	0	480 (8.7)	70 (3.0)
55–74	575 (48.9)	290 (35.8)	2520 (57.7)	665 (42.5)	3095 (55.8)	955 (40.2)
75–84	550 (46.8)	450 (55.6)	1415 (32.4)	900 (57.5)	1965 (35.5)	1350 (56.8)
All	1,175 (14.8)	810 (10.2)	4,365 (55.2)	1,565 (19.8)	5,540 (70.0)	2,375 (30.0)

^a^All numbers are rounded to the nearest 5

The majority of deaths prevented or postponed 5,930 or 74.9%, occurred in “healthy individuals” in the population. A further 1,985 deaths or 25.1% were attributed to secondary prevention in CHD patients. The majority of all deaths prevented or postponed were in older ages (age 55–84), with only 7.0% of deaths prevented in people < 55 years, because the majority of CHD deaths occur among those aged 55 or older. In addition, the biggest effect of primary prevention was in men with a more evident effect in men aged 55 to 74 years. In women the largest effect of primary prevention was in those aged 75 to 84 years.

### Sensitivity analysis

Even if the maximum and minimum estimates varied, the relative contribution for each sub-population in each analysis remained consistent when the multi-way sensitivity analysis was performed, suggesting reasonably robust estimates (Table 2) [19].

## Discussion

Known cardiovascular risk factors improved in both healthy individuals and in patients with known CHD between 1986 and 2002, with the largest effect on mortality effects coming from primary prevention [1]. In this investigation, we studied the relative contribution of risk factor reductions in patients with known CHD and in individuals in the population without a diagnosis of CHD. We found that reductions in the three major risk factors in patients with CHD (secondary prevention) accounted for one quarter of the deaths prevented or postponed, while three quarters of the mortality reduction occurred in the asymptomatic population. These results are consistent with previous studies in Europe and elsewhere [8, 9].

All risk factor changes were greater among CHD patients than individuals in the population. Cholesterol was the single most important risk factor in both CHD patients and asymptomatic individuals, accounting for 69.2% of all deaths prevented or postponed. Major changes in diet occurred during the study period, mainly a decrease in saturated fat intake which was replaced by more healthy products such as olive oil and canola oil. These changes can largely explain the decrease in total cholesterol by 0.64 mmol/L in the total population (from 6.1 to 5.5 mmol/L), with the largest effect coming from dietary changes, and by 1.6 mmol/L (from 6.8 to 5.2 mmol/L) in CHD patients. Cholesterol change is powerful regardless of whether the decrease is due to dietary intervention or medication, and a relatively small decrease of 0.6 mmol/L lowers the risk of CHD [21]. The larger reduction in CHD patients was chiefly due to statin treatment. In primary prevention, the use of statins was low (<5%), and there is accordingly potential to increase statin treatment in high-risk individuals. Consequently, the largest effect would be a combination of lifestyle changes and increased treatment in high risk individuals.

Hypertension treatment in the population had a limited effect despite the fact that Sweden has a high prevalence of hypertension. Less than one third of patients treated with antihypertensive medications reached the treatment goal (140/90) [20]. The rather small effect on mortality from reductions in SBP may therefore reflect a large number of undiagnosed individuals, under-treatment, and/or poor adherence. The effect on SBP of lifestyle changes such as physical activity and salt reduction has not been reliably quantified but decreasing salt intake can probably partly explain the decrease in SBP.

Smoking prevalence decreased by about 10% in both patients and in the population, although a slightly higher proportion of CHD patients were smokers (21% compared with 19%). Happily, smoking rates are comparatively low in Sweden, which could explain the relatively small effect of reducing smoking on CHD death rates.

When comparing the effect of primary and secondary prevention, three quarters of the CHD mortality reduction was attributable to risk factor improvements, mainly in asymptomatic individuals in the population, rather than secondary prevention in CHD patients. However, even in symptomatic individuals, medication adherence remains suboptimal [22] and risk factor control remains poor in CHD patients [23]. Only a minority of CHD patients are targeted for structured lifestyle changes, and even fewer among elderly patients, where most of the deaths occur. More intense secondary prevention efforts including medication as well as lifestyle changes might therefore have some value. The prevalence of healthy lifestyle behaviors in patients with coronary vascular disease is low [24]. Even so, the biggest gains by far are likely to come from additional policy interventions benefiting the entire population.

## Strengths and limitations

Modeling studies have a number of potential strengths. The best models can integrate and simultaneously consider huge amounts of data from many sources. Explicit assumptions can then be tested by sensitivity analyses [19]. However, modeling studies also have limitations. In the present study, around 10% of the decreased mortality remains unexplained, which could be due to factors not included in the model, such as socioeconomic status. Low socioeconomic status is an independent risk factor for CHD in both men and women, and could be a contributory cause to the observed decrease in mortality [17].

Models are also dependent on the extent and quality of data available. For population risk factors a number of sources were used in order to gain representativeness [1]. When national data was available that was considered to be most representative, which was the case for smoking. For cholesterol and SBP different sources was used for 1986 and 2002. In addition, data was obtained from different parts of Sweden (Northern Sweden and Gothenburg) to minimize possible geographical variations and was weighted by population size in different age and sex groups. However, we might not have identified those at highest risk, but since we are incorporating a large population we might be somehow balancing this.

Population data and hospital discharge registries in Sweden are particularly good and almost 100% complete. For example, the Swedish register of intensive care admissions for heart conditions, known as RIKS-HIA (Swedish Register of Information and Knowledge About Swedish Heart Intensive Care Admissions), covers more than 90% of Swedish hospitals. Since Sweden has almost no private hospitals, the data probably accurately reflect the majority of the Swedish population. This, together with a long tradition of upholding registries and national population surveys, should minimize the problem of making assumptions on less reliable data. However, another potential limitation could be differences between the periods in diagnostic criteria and the quality of recorded causes of death which could explain some of the decrease in CHD mortality rates. Even so, autopsy rates for persons dying out-of-hospital were at least 75% below the age of 65 in 2006, and 53% for those aged 65–74. Since autopsy rates increased from the beginning of 1990s until 2006 this would not be a significant problem [25].

The model included only those aged 25–84 years, which excludes more elderly people. Since about 20% of CHD events occur in those aged over 84, the benefits of secondary prevention may be slightly underestimated.

Another limitation is that the model we used is not based on recent data, although there is a continuing decrease of CHD mortality in Sweden and elsewhere [2, 26]. In addition, there is no reason to believe that there have been any major changes prescription rate and medical treatment in people with hypertension in the population [27]. However, there has been an increasing use of statins in “healthy individuals” in the population after 2002 [28]. A more recent model investigating the ongoing reduction in CHD mortality after 2002 would probably have found a greater proportion attributable to statin use.

The effects of the various interventions are calculated from large databases, meta-analyses and clinical trials, with effects that should remain applicable also in a recent setting. Our model, accordingly, should provide useful information about the relative contribution about the effect of interventions on risk factors in a contemporary population. Finally, although we used the best evidence available from statistical reasoning and meta-analysis, there is still a small possibility of publication bias.

## Conclusion

While more than two thirds of the large fall in CHD mortality in Sweden is explained by changes in major cardiovascular risk factors, primary prevention had a four-fold larger effect on mortality than secondary prevention in CHD patients. As even small changes in population risk factors have a large effect on CHD mortality, policy interventions benefiting the entire population should consequently be emphasized. In addition, secondary prevention after CHD is important and patients should be targeted for a more intense follow-up, including both lifestyle changes and optimized medical treatment. Such interventions would be beneficial and consequently lead to further reductions in mortality.
